# Evidence versus expectancy: the development of psilocybin therapy

**DOI:** 10.1192/bjb.2023.28

**Published:** 2024-04

**Authors:** James J. Rucker

**Affiliations:** 1 Institute of Psychiatry, Psychology & Neuroscience, King's College London, UK; 2 South London and Maudsley NHS Foundation Trust, London, UK

**Keywords:** Psilocybin, individual psychotherapy, depressive disorders, psychedelics, clinical trials

## Abstract

Although the development of psilocybin therapy has come as a surprise to many, modern research with the drug has been ongoing for 25 years. Psilocybin therapy is composed of psilocybin dosing sessions embedded within a wider process of psychoeducation, psychological support and integration. Early phase clinical trial evidence is promising, particularly for treatment-resistant depression. However, masking probably fails and expectancy effects may be a part of the mechanism of change. Disambiguating between drug and expectancy effects is a necessary part of the development process, yet this is difficult if masking fails. Hitherto, masking and expectancy have not been routinely measured in psilocybin or other medication trials. Doing so represents an opportunity for research and may influence psychiatry more widely. In this opinion piece I summarise the clinical development process of psilocybin therapy thus far, discussing the hope, the hype, the challenges and the opportunities along the way.

## What is psilocybin therapy?

Psilocybin therapy is a process of intermittent drug sessions with psilocybin, alongside psychological education, support and integration, all delivered in a clinic setting. A similar process of treatment existed prior to 1971 using lysergic acid diethylamide (LSD) (which has a very similar mechanism of action), with some evidence of useful clinical outcomes in a variety of non-psychotic mental health problems.^1–6^ Clinical use of LSD therapy ceased in the UK after the 1971 Misuse of Drugs Act made its routine clinical prescription illegal. Research resumed, tentatively, with early phase studies of psilocybin in humans published from around 1997.^7^

Regardless of the drug, the therapeutic emphasis is on the process of drug sessions alongside a process of psychological support. It may be a new paradigm of treatment in contemporary psychiatry (albeit one with its roots in history). If it is, then it is different from much that has gone before. To re-emphasise, this is not just another ‘drug treatment’. It is, rather, ‘drug-assisted therapy’. The purpose of this article is to describe the current state of development of psilocybin therapy and its possible future within psychiatry, and comment on the opportunities and challenges along the way.

Psilocybin therapy includes significant psychological support around the drug itself. For example, in a recently published protocol for a randomised controlled trial (in which I am involved) of psilocybin therapy in treatment-resistant depression (TRD) that is typical of the field, six sessions of psychological therapy are offered around a single dosing session with psilocybin.^8^ The dosing session itself is supervised by a trained psychological therapist.

Despite the amount of psychological support, much focus is placed on psilocybin as the main agent of therapeutic change, rather like classic antidepressants. This approach is too reductive to be useful here. Psilocybin, like LSD and other psychedelics, makes those under its influence sensitive to context (setting) at the same time as disturbing and amplifying elements of the psyche (set).^9^ A hypothesis, therefore, is that therapeutic efficacy may arise within the interplay of set, setting and drug. This challenges existing paradigms of trial design that seek to separate out drugs from the contextual elements that may bias an accurate assessment of the safety and efficacy of the drug itself. Put another way, by subjecting psychedelic drugs to the same trial designs as other drugs, one may risk throwing the baby out with the bathwater.

## Masking and expectancy

‘Not everything that can be counted counts. Not everything that counts can be counted’ (William Bruce Cameron, 1969)^10^

Clinical trials of psilocybin therapy in a variety of mental health problems are ongoing and, as required for licensing, these follow established paradigms of design that seek to separate out the efficacy of psilocybin itself from confounding contextual factors. This is done by randomising the allocation of participants to groups and implementing allocation concealment in an attempt to mask (‘blind’) participants and the trial team.^8^ The problem here is intuitively obvious: it is almost impossible to mask participants and therapists to allocation in trials using psilocybin, where the drug effect is usually clear to both.

Unmasking is probably extensive. In a two-arm ‘double-blind’ study published in 2022 of two sessions of psilocybin therapy in alcohol use disorder, in which the control arm received diphenhydramine to confuse beliefs about allocation, 93.6% of participants correctly guessed their allocation in the first session, with a mean certainty of 88.5%. In the second session, 94.7% guessed their allocation correctly, with a mean certainty of 90.6%. Study therapists correctly guessed allocation in 92.4% of first sessions and 97.4% of second sessions.^11^

Some methods may reduce the chance of unmasking and the effects of it. For example, in the largest clinical trial of psilocybin therapy in patients to date, 233 participants with TRD were randomised to receive a single dosing session with either 1 mg (subperceptual), 10 mg (perceptual, obvious) or 25 mg of psilocybin (perceptual, more intense).^12^ All received psychological support. Universal allocation to a psilocybin condition is likely to confuse participants’ and trial teams’ beliefs about dose allocation, and further confusion is introduced by using two doses with obvious perceptual effects. Masked, geographically independent raters collected primary outcome data (MADRS scores). This should help to minimise observer expectancy bias (although not against participant expectancy bias). However, the success of masking was not measured in this trial.

A dose-dependent effect of psilocybin on depression scores was observed in this trial. All groups improved, but those who received 25 mg of psilocybin demonstrated statistically (and clinically) significantly larger improvements than those who received 1 mg, with the 10 mg group in between. Statistical separation between the 25 mg dose and 1 mg dose lasted for 6 weeks. A strong placebo effect was observed in the 1 mg group, as is observed in other antidepressant trials.

This result can be interpreted in different ways. A dose-dependent effect in a trial where all received psychological support could be interpreted in favour of a drug-specific effect. However, it could also be interpreted as a dose-dependent expectancy effect in which the more intense the subjective effects were, the more likely the participants would believe they were going to improve. Without further data, it is impossible to speculate further, which illustrates the basic problem.

There is no infallible way around the masking issue (unless one anaesthetises participants before and during dosing). Since this is impractical, unmasking will occur and expectancy effects are … expected.

If so, it represents an opportunity to research expectancy effects. But this is not altogether straightforward. Measuring masking is easy, as you just ask people about their confidence that they received X, Y, Z, etc. Measuring (and modelling) expectancy effects, on the other hand, is rather more challenging.^13,14^ It is possible to design questions that ask participants to rate the ‘extent’ of the expectancy effects they are aware of at different time points and in different scenarios (placebo versus psilocybin, for example). However, this will provide a rough measure only of conscious expectancy. Subconscious expectancy may be more important, but is inherently unmeasurable. Expectancy effects will naturally vary over time and according to other factors (differences between the delivery of psychoeducation, for example), so snapshot measurements will miss important nuance. This is before we consider whether the drug itself is in a form of direct pharmacological interplay with expectancy effects during the dosing session. Tricky indeed.

Even so, it seems to represent a fertile opportunity for research. If masking fails so comprehensively and expectancy effects are expected, here is an opportunity to investigate them. We may discover, for example, that certain contextual factors tend to influence expectancy positively or negatively, thus allowing an optimisation of context. Or that pre-existing characteristics predict positive or negative expectancy. Depending on what we discover, the insights might go further than just psilocybin therapy.^15,16^

A counter position is that if expectancy is so difficult to capture, such research is unlikely to lead to results that change practice, and it risks confusing an already complicated picture.^13^ Further, there are many accepted treatments today where it was inherently impossible to mask participants in trials, and expectancy effects were an inevitable part of the outcome. Physiotherapy, psychotherapy and surgery are good examples. Further, the same phenomenon presumably applies to many drug treatments too, especially in psychiatry, even if it is not routinely measured. For example, the success of masking in the pivotal licensing trials of olanzapine was not reported,^17–20^ but few would expect it to have been adequate given the side-effect profile of the drug.

The effects of such debates are played out in broader spheres than this. Perennial arguments about the efficacy of antidepressants seem endless, partly because evidence about masking and expectancy has not been routinely collected.^16^ Overall, it seems likely that psilocybin trials, because masking seems to fail so reliably, highlight the issue of expectancy effects sufficiently for it to get some airtime in a field that has hitherto shied away from it. This can be seen as both an opportunity for insights into therapeutic development and a problem for regulators making a licensing decision. My own view is that if we can try to measure expectancy, then we probably should. Will this resolve arguments for and against the use of psilocybin therapy (or antidepressants)? Most probably not, but we may discover new, useful knowledge about the role of context and expectancy along the way.

Capturing clinical change in mental health conditions remains difficult. Without objective biomarkers, the field has long relied on subjective scales, and here also lies the potential for bias and confound. The most used and accepted scale for depression severity is the clinician-rated Montgomery–Åsberg Depression Rating Scale (MADRS).^21^ This was designed in 1979 as a scale sensitive to changes in response to classic antidepressants. It is routinely used in psilocybin therapy trials, but psilocybin is not a classic antidepressant. If this is a different approach of therapy, then are we capturing adequately what is changing?^22^ We do not know, but the informal feedback from participants in trials is that classic antidepressant scales do not capture the varied change elicited by psilocybin very well. My own observation is that psilocybin elicits change in rather mysterious and unpredictable ways and can often be the start of a much longer process of coming to terms with the psychosocial drivers of chronic depression. It seems to be ‘antidepressant' in a rather different way to that with which we are familiar.

A variety of scales have been developed to attempt to capture elements of the change observed in early phase trials with psilocybin. ‘Emotional breakthrough’,^23^ ‘mystical’ experience^24^ and the altered states of consciousness experienced in the acute phase of the drug^25^ are some examples. There are many others. Such elements may act as mediators of change,^26^ but since they are not diagnosis specific they have little utility for regulatory purposes.

We might, therefore, be better to keep things simple. A team at Maudsley Hospital have recently developed a very quick and simple visual analogue measure of depression that concentrates on the three core symptoms that are meaningful to patients: mood, experience of pleasure, and suicidality.^27^ The scale can be quickly and repeatedly administered. This can capture both the rapid effect seen with psilocybin, and the variability in response over time that is so common in depression anyway. The advantage of this is that it enables more accurate ‘area under the curve’ analyses of response. Qualitative data are important too.^28^ Such data add narrative colour to quantitative change, informing therapeutic process development and, perhaps, hinting at how change with psilocybin therapy should be measured. Meanwhile, *in vivo* and *in vitro* laboratory work informs on basic mechanism.^29^

It is only with a converging evidence base, replicated over time, that we will begin to form an informed opinion about psilocybin therapy, be it positive or negative. Elements are likely to remain opaque, but at least it will be an opinion formed on observation and data, not the rhetoric and stigma that have marked 50 years of prohibition.

## Hype versus hope

**Fig. 1:**
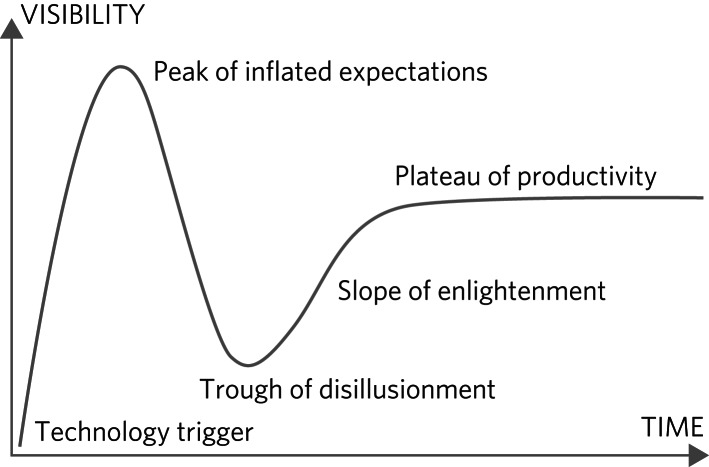
The Gartner hype cycle (original by Jeremy Kemp: https://commons.wikimedia.org/wiki/File:Gartner_Hype_Cycle.svg). Relabelled and used under license CC BY-SA 3.0 (http://creativecommons.org/licenses/by-sa/3.0/).

The general excitement about psilocybin therapy (also a form of expectancy effect) has been described as a bubble, and more recently as a process similar to the ‘Gartner hype cycle’ (Fig. 1).^30^ The idea is that the current ‘hyped hope’ will inevitably lead to ‘disappointed disillusionment’ when the striking (but clinically non-credible) effect sizes reported in early phase, single-centre clinical trials of psilocybin therapy are tempered by later phase research.

It is without doubt that psilocybin therapy is over-hyped. A look at the trajectory of stock prices in the area suggests a correction is well underway. But the hype cycle is not specific to the development of psilocybin, even if the sociopolitical narrative around it may exaggerate the effect. I suspect that the underlying concern is that the field will spin out of control, as it did in the 1960s. This seems unlikely. Modern regulations surrounding safe custody of psychedelics and the conduct of clinical trials are effective and baked in. They allow us to gather evidence in a way that governments (and most of the public) find acceptable. They have the effect of moderating the worst excesses of evangelism and demonisation.^31^ Outside the sphere of medical regulation, where unregulated psychedelic experience ‘retreats’ have sprung up, the outcome could be a different matter.^32^ We should be mindful not to conflate the two.

Where are we now? Twenty-five years since the first modern study of psilocybin in humans,^7^ psilocybin therapy is now on the threshold of phase 3 trials for TRD, due to start in 2023. Phase 3 trials for TRD are (thus far) predicated on the single large multi-centre phase 2b trial mentioned above, which randomised 233 participants with TRD into three groups given a single dosing session with 1 mg, 10 mg or 25 mg of psilocybin, alongside psychological support.^12^ The primary outcome was the MADRS change from baseline at week 3. Results showed that the 25 mg group significantly improved relative to the comparator group, with an effect that was statistically significant until week 6 and clinically significant (−6.6 points on the MADRS, relative to 1 mg) at week 3. The 10 mg group improved relative to the 1 mg group, but not significantly. This trial also highlighted a potential safety signal, with nominally increased suicidal behaviour in the 25 mg group relative to the comparator groups. The aetiology of this remains unclear, although in essence it appears somewhat similar to the phenomenon seen with other licensed treatments for depression. Overall, these results tempered the large effect sizes seen in earlier phase, single-centre trials, although providing a credible basis for phase 3 trials. As I have discussed above, the effects of unmasking and expectancy were not measured in this trial.

In summary, so far we have established what psilocybin therapy is and that the initial clinical evidence for it in TRD is quite promising. However, we have established also that there are difficult problems with masking that make it unclear what is driving this promise. Few have measured masking, and when they have it has failed extensively. Thus, positive expectancy effects associated with receiving psilocybin (as well as negative expectancy associated with receiving placebo) are likely, particularly given that those who are suspicious of psilocybin therapy are unlikely to volunteer for a clinical trial. Measuring expectancy effects seems sensible. However, the picture is more complicated still because psychedelics themselves are likely to be pharmacological moderators of the very expectancy effects we seek to measure. Thus, even if we measure expectancy, what clinical change is driven by pre-existing expectancy effects and what is driven by the drugs’ effects on these would not be possible to discern without further, more complex measurements. Overall, we should not get too caught up in the details, focus on what is practical to measure and keep William Bruce Cameron's quote in mind (and an open mind) as we try to interpret the findings.

## Patient and clinician attitudes

Perhaps a more pertinent question is whether the public (and clinicians) want this research or this treatment. There is some evidence here. In a YouGov survey commissioned by the charity Drug Science in 2020, 1763 adults in the UK were asked about their attitudes towards psilocybin therapy. Respondents were asked ‘Imagine that you personally were suffering from a medical condition where there was strong evidence that a magic mushroom-based treatment (psilocybin-assisted therapy) could be effective. In this scenario, how likely would you be to consider this as a treatment option?’ Fifty-nine per cent indicated they ‘definitely’ or ‘probably’ would consider it.^31^ Fourteen per cent would not (the rest were unsure). Respondents were asked ‘To what extent would you support or oppose the government relaxing restrictions on research into the medical use of magic mushroom-based treatments (psilocybin assisted therapies) for mental health conditions if this didn't affect how it was classified in criminal law (e.g. as a class A drug)?’ Fifty-five percent indicated that they ‘strongly’ or ‘somewhat’ supported that statement. Thirteen per cent were opposed. Conservative voters (51%) were less likely to be supportive than Labour voters (60%) or Liberal Democrat voters (64%). There was little geographical variation.

In a survey of 3050 adults living in the USA, Canada, UK, France and Germany, 65% of respondents agreed or strongly agreed with the statement ‘If faced with a medical condition for which a psychedelic medicine was shown to be safe and effective, I would consider using this treatment option’. One-third agreed or strongly agreed with the statement ‘I know someone who would benefit from psychedelic therapy’.^33^ Forty-three percent of UK respondents agreed or strongly agreed with the statement ‘I support the legalisation of psychedelics for medicinal use’. Fifty-three percent agreed or strongly agreed with the statement ‘I know someone who would benefit from psychedelic therapy’.

In a US survey of 324 psychiatrists, 42.5% moderately or strongly agreed that ‘the use of hallucinogens shows promise in treating psychiatric disorder’. However, 64.9% moderately or strongly agreed that ‘the use of hallucinogens increases the risk for subsequent psychiatric disorders’.^34^

These figures seem to illustrate the extensive divergence of current government policy from public opinion, but also speak to a paradox that is not easy to reconcile: psychedelics are considered to be capable of both help and harm simultaneously. This brings us back to the role of context and expectancy in moderating outcome.

If context and expectancy are important, we should further consider potential confounds of clinical trials with psilocybin. In clinical trials we conduct at The Psychoactive Trials Group, most participants express their interest spontaneously. Although we require that participants must then be referred by their healthcare practitioner to be considered, here may be a significant form of selection bias. Particular people tend to volunteer for trials, and particular people probably tend to volunteer for psillocybin trials. The risk is that samples from such trials may over-emphasise benefit. On the other hand, researchers are ethically bound not to recruit those who are severely unwell. This means placebo response rates are liable to be inflated, diluting the capacity to detect treatment effects.^35^ Such effects on placebo response rates are liable to change, however, according to the varying success of masking. So, the situation is somewhat complex, and the overall message is that we should treat clinical trial results with psilocybin therapy with caution.

One final point of interest (which was raised by a peer reviewer of this piece) is that of seeking informed consent for psilocybin therapy. On the one hand it could be argued that this is a drug like any other. On the other hand it can be argued that this is a drug unlike any other! The psychiatrist Stanislav Grof, who gave LSD psychotherapy for many decades, described the function of psychedelics like psilocybin as ‘to activate the psyche and mediate emergence of the unconscious and superconscious contents into consciousness’.^9^ The problem with seeking informed consent, then, is that neither the participant nor the researcher can know in advance the contents of the participant's subconscious, so technically the consent cannot be ‘informed’.

I tend to take a pragmatic approach here. Consent in clinical trials is inherently on the basis that the trial is an experiment and thus the outcome is uncertain. For psychedelic drugs, there is a further element of uncertainty inasmuch as one may be exposed to the darker throes of the subconscious. Or not. The question for each participant then may be along the lines of ‘do you consent, knowing that this might be a possibility?’, having spoken about what the potential impact might be. It is hard to see what more can be done.

The same issue will be present for psilocybin therapy if it is ever licensed. But I think the same principle probably applies. The same problem, after all, implicitly exists for those forms of psychotherapy where the emergence of subconscious process is understood to be part of the process of change. I reflect, in any case, on the degree to which we are ever really able to fully inform our patients about the effects of psychiatric drugs where we have only the slightest glimmer of insight into their mechanism of action. Psychedelic drugs are special in this regard, but not that special.

## Risks

Psilocybin itself is remarkably non-toxic from a physiological perspective. It does not significantly affect physiological systems that underpin vital functions, because it has no significant activity at neurotransmitter systems other than serotonin.^36^ Even here it is a partial agonist, and has limited activity at the serotonin transporter, so the risk of serotonin syndrome is negligible (although not zero).^36^ The ratio of the toxic dose to the usual dose (estimated at around 1000) suggests it is almost impossible to overdose on.^37^ It has no physical dependence potential and there is no withdrawal syndrome with repeated use.^38^ It has no significant effect on cardiac conductivity and there is no sign of renal or hepatic impairment.^39^

The main risk is from psychological toxicity, and this is likely to be context dependent. When LSD was first discovered, it was labelled, among other things, a ‘psychotogen’. This makes some sense. Classic psychedelics are partial agonists at the 5-HT_2A_ receptor^40^ and many antipsychotic drugs (and antidepressants) are antagonists here. This said, drugs that are only 5-HT_2A_ receptor antagonists do not have clinical antipsychotic effects and there are many examples of antipsychotic drugs (haloperidol and amisulpride, for instance) that show no clinically significant activity at 5-HT_2A_. And yet, classic psychedelics elicit some of the positive symptoms of acute psychosis – misperceptions, ego dissolution, blurring of conceptual boundaries – albeit restricted to the acute phase in the vast majority.

Such changes do not appear to be associated with harm, at least in medical settings. A recent exploratory trial showed that a single 25 mg dose of psilocybin delivered in a clinical research setting did not lead to negative (or positive) changes in a variety of cognitive and emotional processing measures when compared with placebo.^41^ Pre-prohibition trials giving LSD to those with chronic psychoses observed that it worsened symptoms in the acute phase, before patients generally returned to baseline.^42^ Observational population studies of drug use and mental health outcomes do not find an association between psychedelic use and the emergence of psychotic disorders.^43^ In contrast, such analyses find that classic psychedelic use is associated with reduced suicidality and use of psychiatric medication.^44^ It is likely, however, that there are many confounds here.

Cases of psychosis, mania, suicide or homicide in association with psychedelic drug use have occurred and will occur. Whether such drug use is coincident or causative is usually shrouded in complexity. Beyond the drugs themselves are the obvious psychosocial disadvantages that correlate with criminalised use of drugs in the first place. It seems likely that the media will continue to focus on rare, attention-grabbing horror stories involving psychedelics. But we should be mindful of the reporting bias that exists here.

**Fig. 2:**
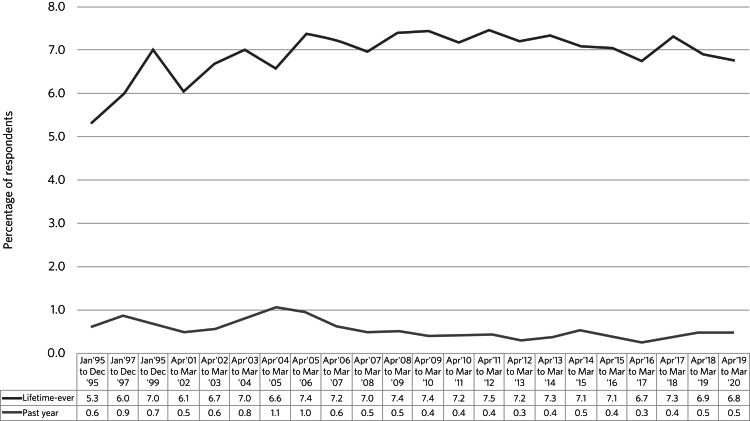
Percentage of respondents reporting lifetime-ever or past year use of psilocybin mushrooms in the UK National Crime Survey from 1995 to 2020.^45^

A broader concern is that clinical trials with psychedelics will drive non-medical use. So far, this is not reflected in UK data. Figures from the National Crime Survey for 2020 show that 6.8% of 16- to 59-year-olds reported lifetime-ever use of psilocybin mushrooms, with 0.5% reporting past year use.^45^ This figure has been stable since records began in 1995 (Fig. 2). Criminalisation of end users is liable to induce people to lie about use, so rates may be higher than data suggest.

A drive towards medical adoption will probably lead to increased rates of use, but part of this will be attributable to people feeling they can be honest about use that was happening already. Increased transparency about non-medical use should result in increased access to education and harm minimisation advice. Our research shows that rates of seeking emergency medical care in response to recreational psilocybin mushroom and LSD use are, in any event, notably low.^46,47^

Overall, psychedelic drugs have risks like any others. The direct physiological risk of psilocybin is notably low. Psychological toxicity does occur, but is likely to be context dependent. Controlling context in a medical setting should reduce risks. Increased medical use may drive increased reported rates of recreational use, but in part this is likely to be reflective of increased transparency. A more open framework for discussing psychedelics, in turn, should encourage recreational users to access harm minimisation efforts. Rare tragedy is, however, inevitable and it is likely still that the media will choose to focus attention disproportionately here.

## A future

If phase 3 trials of psilocybin therapy for TRD are sufficiently positive and regulators are satisfied that any safety signals are proportionate to the risks of TRD itself, licensing will likely follow.

Psilocybin therapy's nature as a form of ‘drug-assisted therapy’ may have cross-diagnostic utility beyond TRD, extending from anorexia nervosa^48^ to alcoholism.^11^ It may also have potential for use in those whose existing medical conditions preclude other more toxic treatments (even selective serotonin reuptake inhibitors).

Licensing allows prescription, but does not necessarily imply availability. There are some practical hurdles to wider adoption. Can psilocybin therapy be implemented within an existing mental health infrastructure? Who will pay for it? Who will deliver it? How will it be regulated? I will speculate here.

Psilocybin therapy does not need special infrastructure. A cosmetically modified, quiet clinic setting or day hospital will suffice. Who will pay depends on how much it costs. It will be much more expensive than primary care antidepressant treatment. But whether it is more expensive than a 16-week course of cognitive–behavioural therapy, intensive treatments delivered by community mental health teams, a course of electroconvulsive therapy (ECT) or a crisis admission seems more doubtful. Health economic analyses are underway.

Therapists will deliver the psychological support and dosing sessions, with psychiatrists prescribing and monitoring medical aspects. Therapists will need to be trained, and although these programmes have been developed, training at scale is a significant hurdle.

Regulation of therapy delivery is likely to be critical to maintaining safety, quality and sustainability. If context is so important, it will need to be independently scrutinised and assured. A framework that may act as inspiration is the accreditation service for clinics delivering ECT in the UK. Such an accreditation should be compulsory for any centre delivering psilocybin therapy. Finally, psilocybin should be excluded from the compulsory treatment provisions of mental health legislation. There is no place for psilocybin therapy without the ability to give freely informed consent.

To test some of these questions, we have recently begun construction of a dedicated research and treatment centre for psilocybin therapy at King's College London and the South London and Maudsley NHS Foundation Trust.^49^ The first stage is due to open in 2023. The intention is that it acts as a model psilocybin therapy ‘clinic of the future’ at the same time as being a clinical research centre. Such a centre will allow a more precise definition and control of context than in more general clinical research facilities. It will allow the processes and structure of a therapist training programme to be tested. It will allow the feasibility of simultaneous dosing sessions to be tested. In this sense, it is a more accurate test of how psilocybin therapy might work in the real world. If it is successful, it could be a model that others can mimic.

## Conclusions

Although psilocybin therapy is promising, we should remain equipoised. There is hope, but also much hype. Recent results show that psilocybin therapy is not a panacea. Our own observation is that it is not a ‘quick fix’ in most. Rather, it can act as the catalyst of a longer process of reconciliation and engagement with the psychosocial drivers of depression, where ongoing therapy has a nurturing role for any insights gained. In this sense, it is something different from what we already have, and something that appeals to those to whom daily medication treatments do not. It is unlikely to replace any existing treatment. I hope that an emergence of some clarity about how non-drug factors are mediators of outcome will stimulate a more nuanced conversation about the role of contextual elements in therapeutic outcome more generally, perhaps informing mental healthcare more widely. The trial data, and the associated mechanism data, should speak for themselves. Then should follow a more reasoned conversation about psilocybin therapy: what it might achieve, and what it will not.

## Data Availability

Data availability is not applicable to this article as no new data were created or analysed in this study.

## About the author

**James J. Rucker**, MBBS, MRCPsych, PhD, is a consultant psychiatrist and senior clinical lecturer in psychopharmacology in the Department of Psychological Medicine at the Institute of Psychiatry, Psychology & Neuroscience, King's College London, and an honorary consultant psychiatrist at the National Affective Disorders Service, Maudsley Hospital, South London and Maudsley NHS Foundation Trust, London, UK.
